# Bridging the Gap: Infantile Hemangioma Care Variability Among Pediatric Care Physicians in Greece

**DOI:** 10.7759/cureus.104076

**Published:** 2026-02-22

**Authors:** Alexios Alexopoulos, Dimitrios Ntokos, Louiza Kontara, Maria Malliarou, Evangelos C Fradelos, Maria Saridi, Aikaterini Toska, Pavlos Sarafis, Lamprini Nasi, Despina Briana

**Affiliations:** 1 First Department of Pediatrics, School of Medicine, National and Kapodistrian University of Athens, Agia Sophia Children's Hospital, Athens, GRC; 2 Department of Surveying and Geoinformatics Engineering, School of Engineering, University of West Attica, Athens, GRC; 3 Department of Pediatrics, North Middlesex University Hospital, London, GBR; 4 Department of Nursing, University of Thessaly, Larissa, GRC; 5 NICU, Third Department of Pediatrics, School of Medicine, National and Kapodistrian University of Athens, Attikon University Hospital, Athens, GRC

**Keywords:** clinical decision-making, guideline implementation, healthcare implementation, pediatric pharmacotherapy, physician survey, practice variation, primary care, propranolol therapy, treatment initiation

## Abstract

Infantile hemangiomas (IHs) are the most common benign vascular tumors of infancy, and timely, risk-adapted management is critical to prevent functional and aesthetic complications. Although international evidence-based guidelines are well established, their implementation in everyday primary care practice remains inconsistent. We conducted a pragmatic, cross-sectional, web-based survey between November 2024 and February 2025 to characterize real-world management patterns of IHs among Greek primary care physicians who had completed a nationally accredited e-learning program on IH recognition and treatment. The survey comprised 10 multiple-choice items addressing treatment initiation criteria, pharmacologic strategies, monitoring and imaging practices, and treatment discontinuation. Seventy-four physicians participated, including 48 pediatricians (64.9%) and 26 general practitioners (GPs; 35.1%). Head and neck IHs larger than 2 cm prompted intervention in 40/48 pediatricians (83.3%) and 24/26 GPs (92.3%), compared with significantly lower intervention rates for similarly sized lesions on the trunk or extremities (30/48, 62.5% vs. 10/26, 38.5%; p = 0.048). Ulceration was infrequently recognized as an independent indication for systemic therapy (12/48 pediatricians, 25.0%; 4/26 GPs, 15.4%). Propranolol was widely adopted as first-line treatment; however, initiation most commonly occurred after 12 weeks of age (59/74 physicians, 79.7%), inpatient commencement was strongly favored even in low-risk cases (67/74, 90.5%), and dosing clustered around 2 mg/kg/day (71/74, 95.9%). Treatment discontinuation was typically abrupt (49/74, 66.2%), with structured tapering and post-treatment relapse surveillance infrequently reported. Despite shared theoretical training, substantial variability persists in the real-world management of IHs in Greek primary care, reflecting an implementation gap rather than a lack of knowledge. These findings highlight the need for context-sensitive national guidance emphasizing risk-adapted initiation and dosing, clearly defined imaging thresholds, safe outpatient pathways, and standardized follow-up to improve consistency and equity of care.

## Introduction

Infantile hemangiomas (IHs) are the most common benign vascular tumors of infancy, affecting approximately 5-10% of neonates [1,2]. Their increased prevalence among girls, preterm or low-birth-weight infants, and Caucasian populations is well documented, although the underlying mechanisms remain incompletely understood [3]. Current etiologic models emphasize aberrant angiogenesis driven by the homing of circulating endothelial progenitor cells to hypoxic or developmentally vulnerable tissues, while competing hypotheses-including placental embolization and somatic mutations in angiogenic pathways-underscore the heterogeneity of IH pathogenesis and suggest that no single pathogenic route explains all phenotypes [4-7].

Clinically, IHs typically follow a biphasic course, characterized by a rapid proliferative phase during the first months of life and gradual involution over subsequent years [1,2,8]. Although many lesions regress without significant sequelae, deeper or bulky IHs may result in telangiectasia, fibrofatty residua, or permanent scarring, with potential psychosocial consequences later in life [7,9]. Early risk stratification is therefore critical, particularly for lesions involving the periorbital, perioral, or airway regions, where complications such as visual obstruction, ulceration, hemorrhage, or, more rarely in multifocal disease, high-output cardiac failure may occur [10-13]. In response to these risks, referral to specialized multidisciplinary care by four weeks of age has been recommended; however, adherence to this target in routine clinical practice remains uncertain [10,14].

The therapeutic landscape of IHs was fundamentally reshaped following the recognition of propranolol’s efficacy in 2008, which is now firmly established as first-line therapy for high-risk lesions at doses of 2-3 mg/kg/day [6-8,10,15-17]. Alternative beta-blockers and topical agents may be considered in selected cases, while laser or surgical interventions retain a role in refractory or residual disease [6-8,10,18-21]. Despite detailed position statements issued by European, North American, and Australasian expert groups [9,14,22], considerable variability persists in the real-world implementation of these recommendations.

How international guidelines are interpreted and applied by primary care physicians, particularly in healthcare systems where initial IH assessment often occurs outside tertiary referral centers, remains insufficiently explored. In Greece, where both general practitioners (GPs) and pediatricians play a central role in early IH management, the extent to which standardized theoretical training translates into consistent, evidence-aligned, and implementable clinical practice is unknown. To address this gap, we conducted a national survey among primary care physicians who had completed a unified e-learning program on IH management, aiming to characterize real-world practice patterns, identify areas of divergence between guideline recommendations and implementation, and inform context-sensitive strategies for improving their application.

## Materials and methods

Study design, population, and recruitment

This pragmatic, cross-sectional study was designed to capture a real-world snapshot of clinical practice in the management of IHs among primary care physicians in Greece. The primary objective was to document variability in routine clinical decision-making related to treatment initiation, monitoring, and follow-up, rather than to test causal hypotheses, consistent with an implementation-oriented approach to guideline adherence. Data were collected prospectively using a web-based, forward-looking survey design, without accessing medical records or patient-level clinical data.

Eligible participants were licensed primary care physicians who self-identified as either GPs or board-certified pediatricians, were involved in the care, referral, or follow-up of infants with IHs, and had completed a nationally accredited e-learning course on IH diagnosis and management in 2023. The course curriculum was based on contemporary European and Australasian recommendations and served as a standardized inclusion criterion to ensure a uniform baseline level of theoretical training [9,14,22].

An initial pool of 150 physicians was identified through publicly available professional registries and verified enrollment records from the national IH e-learning program. Of these, 60 were excluded prior to invitation due to a lack of documented completion of the accredited IH e-learning program or inactive involvement in pediatric primary care. Following eligibility screening and deduplication, 90 physicians met the inclusion criteria and were invited to participate between October 18 and October 30, 2024. Invitations included a brief study description, a GDPR-compliant electronic informed consent form, and a personalized link to the online questionnaire. Non-responders received up to two reminder emails at 14-day intervals. Ultimately, 74 physicians completed the survey (response rate 82.2%), while 16 did not respond. The survey platform required completion of all items prior to submission; therefore, no partially completed questionnaires were included in the final analysis.

The study protocol was reviewed and approved by the Scientific Committee of the Agia Sophia Children’s Hospital, Athens, Greece (approval reference: 4832/12.04.2023). Electronic informed consent was obtained from all participants prior to questionnaire access. Participation was voluntary. The study involved only healthcare professionals and did not include patients or patient-level clinical data. No treatments, medications, or clinical procedures were administered, dictated, or modified as part of the study. The study was conducted in accordance with the principles of the Declaration of Helsinki and the CHERRIES guidelines for reporting internet-based surveys.

Survey instrument, data collection, and data security

Data were collected using a structured, web-based questionnaire consisting of 10 multiple-choice items, with multiple responses permitted where appropriate to reflect the multifactorial nature of IH-related clinical decision-making. The questionnaire covered four core domains of clinical management: treatment initiation criteria, pharmacologic strategies, monitoring and safety practices, and treatment discontinuation with relapse surveillance. A detailed overview of survey items and response options is provided in Table 1 and informed the presentation of results.

**Table 1:** Survey instrument Structure and content of the questionnaire administered to primary care physicians, outlining the clinical domains assessed in the management of infantile hemangiomas.

ID	Survey question
Q1	Which patients with infantile hemangiomas require treatment?
Q2	At what age should propranolol treatment be initiated, if indicated?
Q3	Is propranolol prescribed as a generic or brand-name formulation?
Q4	What is the recommended propranolol dosage for infantile hemangiomas?
Q5	What is the recommended duration of propranolol therapy?
Q6	Should propranolol be initiated on an outpatient basis or require hospitalization?
Q7	How frequently should patients receiving propranolol be monitored?
Q8	Which patients require additional investigations (e.g., ECGs or ultrasound)?
Q9	What is the recommended method for discontinuing propranolol therapy?
Q10	Is follow-up required after discontinuation of propranolol treatment?

Content validity was assessed through blinded expert review by two pediatric dermatologists and one health services researcher with experience in IH management and peer-reviewed publications in the field [6,8,19,23]. Pilot testing with five non-participating primary care physicians resulted in minor linguistic refinements without structural modification. Formal psychometric testing was not undertaken, given the descriptive and exploratory nature of the study.

The questionnaire was administered via a GDPR-certified online platform (LimeSurvey v3.28.2). Mandatory response settings prevented item omission, while IP masking and SSL encryption ensured respondent anonymity. No personally identifiable information was collected beyond age category and years of clinical practice. Data were exported within 24 hours of survey closure on February 28, 2025, and stored on an encrypted, access-restricted institutional server. Multiple responses were permitted for selected items; therefore, aggregated percentages may exceed 100%.

Statistical analysis

Statistical analyses were performed using IBM SPSS Statistics for Windows, Version 29 (Released 2023; IBM Corp., Armonk, New York, United States). Descriptive statistics, including frequencies (n) and percentages (%), were used to summarize survey responses. Comparisons between pediatricians and GPs were conducted using Pearson’s chi-square test or Fisher’s exact test, as appropriate. Statistical significance was set at α = 0.05.

For chi-square analyses, test statistics (χ² values with degrees of freedom, df = 1) and effect sizes were quantified using Cramér’s V. For comparisons evaluated using Fisher’s exact test, effect estimates were expressed as risk differences (RD) with corresponding 95% confidence intervals. Given the exploratory nature of the study and the modest sample size (n = 74), multivariable modeling was not performed.

## Results

Respondent characteristics and treatment initiation patterns

Of the 90 primary care physicians invited, 74 completed the survey, yielding a response rate of 82.2%. Respondents included 48 pediatricians (64.9%) and 26 GPs (35.1%), reflecting the shared contribution of both specialties to pediatric primary care in Greece. For selected questionnaire items allowing multiple responses, percentages represent the proportion of respondents selecting each option.

Despite uniform exposure to the same nationally accredited e-learning module on IHs, substantial variability in clinical decision-making was observed. Decisions regarding treatment initiation versus observation differed according to lesion size, anatomical location, morphology, and perceived functional risk. Propranolol was identified as the first-line treatment of choice by the vast majority of respondents and was predominantly prescribed in its brand-name formulation, with no statistically significant difference between pediatricians (38/48, 79.2%) and GPs (22/26, 84.6%; Fisher’s exact test, p = 0.740; Table 2).

**Table 2:** Reported management practices for infantile hemangiomas Distribution of survey responses among pediatricians and general practitioners regarding treatment initiation criteria, pharmacologic strategies, monitoring and imaging practices, treatment discontinuation, and post-treatment follow-up. Data are presented as number (n) and percentage (%). Group comparisons were performed using Pearson’s chi-square test or Fisher’s exact test, as appropriate, depending on cell counts. For chi-square analyses, test statistics (χ², df = 1) and effect sizes are reported as Cramér’s V. For Fisher’s exact test comparisons, effect size is expressed as risk difference (RD) with 95% confidence intervals. Multiple responses were permitted for selected questions; therefore, percentages may exceed 100%. A two-sided significance level of 0.05 was applied throughout.

Survey question (Q)	Clinical domain	Response option	Pediatricians (n = 48)	General practitioners, GPs (n = 26)	Risk difference (RD)	Standard error (SE)	95% CI of RD		p-value	χ² (df=1)	Effect size	Statistical test
							Lower	Upper				
Q1	Treatment criteria	Head/neck IH > 2 cm	40 (83.3%)	24 (92.3%)	-9.0%	0.075	-23.7%	5.7%	0.478	-	-	Fisher
		Trunk/extremities IH > 3 cm	30 (62.5%)	10 (38.5%)	24.0%	0.118	0.9%	47.2%	0.048	3.92	0.23	Pearson
		Ulceration	12 (25.0%)	4 (15.4%)	9.6%	0.094	-8.9%	28.1%	0.337	0.92	0.11	Pearson
		Functional impairment (periocular, lip, nasal, airway)	46 (95.8%)	18 (69.2%)	26.6%	0.095	8.0%	45.2%	0.001	-	-	Fisher
Q2	Age at initiation	> 12 weeks of age	40 (83.3%)	19 (73.1%)	10.2%	0.102	-9.8%	30.3%	0.295	1.10	0.12	Pearson
		≤ 12 weeks of age	8 (16.7%)	7 (26.9%)	10.3%	0.102	-30.3%	9.8%	0.295	1.10	0.12	Pearson
Q3	Propranolol formulation	Generic	10 (20.8%)	4 (15.4%)	5.4%	0.092	-12.6%	23.5%	0.740	-	-	Fisher
		Brand-name formulation	38 (79.2%)	22 (84.6%)	-5.4%	0.092	-23.5%	12.6%	0.740	-	-	Fisher
Q4	Propranolol dosage	1–2 mg/kg/day	46 (95.8%)	25 (96.2%)	-0.3%	0.047	-9.6%	9.0%	1.000	-	-	Fisher
		2–3 mg/kg/day	2 (4.2%)	1 (3.8%)	0.3%	0.047	-9.0%	9.6%	1.000	-	-	Fisher
Q5	Duration of therapy	Until 12 months of age	35 (72.9%)	17 (65.4%)	7.5%	0.113	-14.7%	29.7%	0.499	0.46	0.08	Pearson
		< 12 months of age	13 (27.1%)	9 (34.6%)	-7.5%	0.113	-29.7%	14.7%	0.499	0.46	0.08	Pearson
Q6	Treatment setting	Outpatient initiation	6 (12.5%)	1 (3.8%)	8.7%	0.061	-3.3%	20.6%	0.410	-	-	Fisher
		Inpatient initiation	42 (87.5%)	25 (96.2%)	-8.7%	0.061	-20.6%	3.3%	0.410	-	-	Fisher
Q7	Monitoring frequency	Every 12 weeks	16 (33.3%)	13 (50.0%)	16.7%	0.119	-40.1%	6.7%	0.161	1.97	0.16	Pearson
		Every 8 weeks	30 (62.5%)	12 (46.2%)	-16.7%	0.120	-7.2%	39.9%	0.175	1.84	0.16	Pearson
		Every 4 weeks	2 (4.2%)	1 (3.8%)	0.3%	0.047	-9.0%	9.6%	1.000	-	-	Fisher
Q8	Additional investigations	ECGs	34 (70.8%)	24 (92.3%)	-21.5%	0.084	-37.9%	-5.0%	0.032	4.59	0.25	Pearson
		Ultrasound (hepatic/brain) when > 2 IHs	41 (85.4%)	20 (76.9%)	8.5%	0.097	-10.5%	27.5%	0.359	-	-	Fisher
Q9	Discontinuation strategy	Gradual taper (4–8 weeks)	15 (31.3%)	10 (38.5%)	-7.2%	0.117	-30.1%	15.6%	0.531	0.39	0.07	Pearson
		Abrupt cessation	33 (68.8%)	16 (61.5%)	7.2%	0.117	-15.6%	30.1%	0.531	0.39	0.07	Pearson
Q10	Post-treatment follow-up	Yes	8 (16.7%)	4 (15.4%)	1.3%	0.089	-16.1%	18.7%	0.886	-	-	Fisher
		No	40 (83.3%)	22 (84.6%)	-1.3%	0.089	-18.7%	16.1%	0.886	-	-	Fisher

Head and neck IHs larger than 2 cm prompted treatment initiation in 40/48 pediatricians (83.3%) and 24/26 GPs (92.3%), with no statistically significant between-group difference (RD = −9.0%, 95% CI −23.7% to 5.7%; Fisher’s exact test, p = 0.478). In contrast, IHs larger than 3 cm located on the trunk or extremities were more frequently treated by pediatricians (30/48, 62.5%) than by GPs (10/26, 38.5%), a difference that reached statistical significance (RD = 24.0%, 95% CI 0.9% to 47.2%; χ²(1) = 3.92, p = 0.048; Cramér’s V = 0.23).

Ulceration was infrequently recognized as a standalone indication for systemic therapy, reported by 12/48 pediatricians (25.0%) and 4/26 GPs (15.4%), without a significant between-group difference (RD = 9.6%, 95% CI −8.9% to 28.1%; χ²(1) = 0.92, p = 0.337; Cramér’s V = 0.11). In contrast, functional impairment involving periocular, oral, nasal, or airway involvement was widely acknowledged as a trigger for treatment initiation, reported by 46/48 pediatricians (95.8%) and 18/26 GPs (69.2%), with a statistically significant difference favoring pediatricians (RD = 26.6%, 95% CI 8.0% to 45.2%; Fisher’s exact test, p = 0.001).

Treatment setting, monitoring practices, and imaging

A strong preference for inpatient initiation of propranolol was observed across both groups. Hospital-based initiation was reported by 42/48 pediatricians (87.5%) and 25/26 GPs (96.2%), with no statistically significant difference between specialties (RD = −8.7%, 95% CI −20.6% to 3.3%; Fisher’s exact test, p = 0.410).

Treatment initiation most commonly occurred after 12 weeks of age, reported by 40/48 pediatricians (83.3%) and 19/26 GPs (73.1%), with no significant difference between groups (RD = 10.3%, 95% CI −9.8% to 30.3%; χ²(1) = 1.10, p = 0.295; Cramér’s V = 0.12). Target dosing clustered around 2 mg/kg/day, typically administered in two divided doses with gradual dose escalation, selected by 46/48 pediatricians (95.8%) and 25/26 GPs (96.2%), without a between-group difference (Fisher’s exact test, p = 1.000).

Monitoring and imaging practices varied. Electrocardiography was more frequently reported by GPs (24/26, 92.3%) than pediatricians (34/48, 70.8%), a difference that reached statistical significance (RD = −21.5%, 95% CI −37.9% to −5.0%; χ²(1) = 4.59, p = 0.032; Cramér’s V = 0.25). Hepatic or cerebral ultrasound in the presence of more than two cutaneous IHs was reported by both pediatricians (41/48, 85.4%) and GPs (20/26, 76.9%), without a statistically significant difference (RD = 8.5%, 95% CI −10.5% to 27.5%; Fisher’s exact test, p = 0.359). Although the use of cardiac investigations was not directly queried, the high rate of inpatient initiation suggests that baseline cardiac evaluation, including electrocardiography and possibly echocardiography, was commonly incorporated into baseline assessment.

Dosing strategies, treatment discontinuation, and follow-up

With regard to the dosing strategy, most respondents reported targeting 2 mg/kg/day of propranolol, consistent with commonly cited international practice ranges. Treatment duration was relatively uniform, with propranolol discontinued at approximately 12 months of age by 35/48 pediatricians (72.9%) and 17/26 GPs (65.4%), without a significant difference between groups (RD = 7.5%, 95% CI −14.7% to 29.7%; χ²(1) = 0.46, p = 0.499; Cramér’s V = 0.08).

Discontinuation practices were predominantly unstructured. Abrupt cessation of propranolol was reported by 33/48 pediatricians (68.8%) and 16/26 GPs (61.5%), while gradual tapering over 4-8 weeks was reported by 15/48 pediatricians (31.3%) and 10/26 GPs (38.5%), with no statistically significant differences between groups (χ²(1) = 0.39, p = 0.531; Cramér’s V = 0.07).

Post-treatment follow-up after propranolol discontinuation was infrequently reported, with only 8/48 pediatricians (16.7%) and 4/26 GPs (15.4%) indicating routine follow-up (RD = 1.3%, 95% CI −16.1% to 18.7%; Fisher’s exact test, p = 0.886).

## Discussion

Variability in IH management despite standardized training

Despite decades of accumulated clinical experience and the availability of structured international guidelines, the management of IHs remains heterogeneous. The present findings demonstrate that this variability persists even among physicians who have completed standardized training in IH recognition and treatment, underscoring a persistent gap between theoretical alignment and real-world implementation. Similar patterns have been described across other areas of pediatric dermatology and guideline-driven care, where evidence-based recommendations are variably interpreted in everyday practice [4,9,14,22].

Within Greek primary care, this heterogeneity manifests in divergent thresholds for treatment initiation and monitoring. Decisions were frequently influenced by lesion visibility, anatomical location, and anticipated disfigurement rather than by uniform risk-based criteria. While individualized clinical judgment is an essential component of pediatric care, the absence of clearly operationalized thresholds may contribute to delayed intervention, inconsistent treatment patterns, and inefficient use of healthcare resources. Notably, ulceration, despite its established association with pain, infection, and scarring, was under-recognized as a standalone indication for systemic therapy, in contrast to current expert recommendations [8,10-12,14]. These findings suggest not a lack of knowledge, but an interpretive drift whereby clinicians adapt guideline principles to local constraints, perceived risks, and experiential heuristics.

Between consensus and practice: key points of divergence

Since the introduction of propranolol as first-line therapy, the therapeutic landscape of IHs has been fundamentally reshaped. Robust evidence has established its efficacy and favorable safety profile compared with historical alternatives, consolidating its role as the standard of care for high-risk lesions [15,16,24]. Nevertheless, several dimensions of IH management remain inconsistently applied in everyday practice.

One major axis of divergence concerns the dosing strategy. Although international guidelines endorse a range of 2-3 mg/kg/day, most respondents favored the lower end. This conservative preference may reflect safety concerns; however, emerging evidence suggests that higher dosing can achieve superior lesion regression without a proportional increase in serious adverse events [17,25-27]. In the absence of stratified recommendations based on lesion morphology, age, or anatomical site, dosing decisions remain largely discretionary.

Timing of initiation represents a second area of misalignment. Propranolol demonstrates maximal benefit during the proliferative phase of IHs, with early initiation associated with improved functional and aesthetic outcomes [28,29]. In the present cohort, treatment was commonly initiated after this critical window, potentially reflecting referral delays, limited prescribing confidence in early infancy, or structural barriers within primary care. Increasing evidence supporting the safety of propranolol in very young infants further challenges traditional caution and highlights the need to recalibrate initiation thresholds [9,22].

Imaging practices constituted a third point of divergence. While cardiac imaging is indicated in suspected PHACE syndrome [14], its broader use in low-risk infants suggests precautionary rather than evidence-driven decision-making. In our cohort, electrocardiography was more frequently reported by general practitioners than by pediatricians, despite the absence of clear risk indicators in most cases. Notably, this difference corresponded to one of the largest effect sizes observed in the study (Cramér’s V = 0.25), underscoring a clinically meaningful divergence rather than a marginal statistical finding.

Similarly, hepatic ultrasound was frequently employed in infants with limited cutaneous involvement, despite contemporary guidance recommending imaging only in the presence of multiple lesions or systemic signs [17,24,25]. Such precautionary imaging practices may contribute to unnecessary investigations, increased parental anxiety, and inefficient use of healthcare resources, without demonstrable clinical benefit.

Implementation gaps and implications for practice refinement

The patterns observed in this study reflect not only random variation but also systemic implementation gaps. A particularly salient example is the strong preference for inpatient propranolol initiation, even in infants without high-risk features. Although outpatient protocols have been shown to be safe under defined conditions [5,6,10,11], the absence of locally endorsed pathways likely reinforces conservative, resource-intensive practices.

Discontinuation strategies further illustrate the limitations of existing guidance. Despite evidence of rebound growth in a substantial subset of patients, particularly following abrupt cessation or shorter treatment durations [18,29], structured tapering and post-treatment surveillance were rarely reported. Current guidelines provide limited operational detail on discontinuation logistics, leaving clinicians to rely on institutional norms or individual judgment.

Addressing these gaps requires more than reiteration of consensus statements. Context-sensitive implementation tools, including risk-adapted dosing frameworks, explicit imaging thresholds, outpatient initiation checklists, and standardized discontinuation and follow-up pathways, are needed to translate evidence into routine practice. Future research should integrate implementation science approaches to better understand clinician behavior, institutional constraints, and parental expectations that shape decision-making in primary care settings.

Taken together, these findings suggest that improving IH care requires not only additional knowledge dissemination, but also targeted implementation strategies aligned with the realities of primary care practice. Viewed through this lens, heterogeneity becomes informative rather than problematic, offering a critical entry point for intelligent standardization that respects clinical nuance while reducing unwarranted variation. Understanding how and why divergence occurs is a prerequisite for developing adaptable frameworks that promote consistent, timely, and equitable care across diverse system-level realities.

Limitations

The findings of this study should be interpreted within the context of its design. As a cross-sectional survey, the analysis provides a snapshot of reported clinical practice and does not aim to establish causal relationships or temporal changes in decision-making. Data were self-reported and may reflect perceived rather than directly observed behavior; however, this approach is appropriate for implementation-focused research examining real-world guideline interpretation.

All participants had completed the same nationally accredited e-learning program, ensuring a standardized baseline of theoretical training, although individual clinical experience and local practice environments were not formally assessed. The survey did not quantify the annual case volume of IHs managed by each physician, nor were responses weighted according to clinical exposure. All responses were analyzed with equal weighting, consistent with the exploratory implementation-focused design. The sample size was adequate for descriptive and exploratory comparisons with effect size estimation but did not support multivariable modeling.

Finally, while the findings reflect the Greek primary care context, the identified implementation gaps are likely relevant to similar healthcare settings where IH management occurs outside tertiary centers. At the same time, the uniform educational background of participants strengthens internal consistency and allows observed variability to be interpreted as implementation-related rather than knowledge-based.

## Conclusions

This national survey demonstrates that, despite shared theoretical training, the management of IHs among Greek primary care physicians remains heterogeneous. Although propranolol is widely adopted as first-line therapy, substantial variability persists in decisions regarding treatment initiation, dosing, hospitalization, imaging, and follow-up. A substantial proportion of clinicians initiate therapy after the early proliferative phase, favor conservative dosing strategies, and predominantly rely on inpatient protocols, even in low-risk cases. Ulceration is frequently under-recognized as an independent indication for systemic treatment, while discontinuation practices are commonly unstructured and lack systematic relapse surveillance.

Taken together, these findings highlight a clear implementation gap, whereby evidence-based recommendations are inconsistently translated into routine clinical practice. Addressing this gap requires context-sensitive national guidance emphasizing risk-adapted initiation and dosing, clearly defined imaging thresholds, safe outpatient initiation pathways, and standardized discontinuation and follow-up strategies. Such approaches may support more consistent, timely, and equitable care for infants with high-risk hemangiomas.
